# Effects of the energy balance transition on bone mass and strength

**DOI:** 10.1038/s41598-023-42467-6

**Published:** 2023-09-14

**Authors:** Ian J. Wallace, Christopher Toya, Mario Antonio Peña Muñoz, Jana Valesca Meyer, Taylor Busby, Adam Z. Reynolds, Jordan Martinez, Travis Torres Thompson, Marcus Miller-Moore, Alexandra R. Harris, Roberto Rios, Alexis Martinez, Tea Jashashvili, Christopher B. Ruff

**Affiliations:** 1 Department of Anthropology, University of New Mexico, Albuquerque, NM 87131 USA; 2 Natural Resources Department, Pueblo of Jemez, NM 87024 USA; 3 Department of Integrative Anatomical Sciences, Keck School of Medicine, University of Southern California, Los Angeles, CA 90033 USA; 4 Center for Functional Anatomy and Evolution, Johns Hopkins University School of Medicine, Baltimore, MD 21205 USA

**Keywords:** Biological anthropology, Bone

## Abstract

Chronic positive energy balance has surged among societies worldwide due to increasing dietary energy intake and decreasing physical activity, a phenomenon called the energy balance transition. Here, we investigate the effects of this transition on bone mass and strength. We focus on the Indigenous peoples of New Mexico in the United States, a rare case of a group for which data can be compared between individuals living before and after the start of the transition. We show that since the transition began, bone strength in the leg has markedly decreased, even though bone mass has apparently increased. Decreased bone strength, coupled with a high prevalence of obesity, has resulted in many people today having weaker bones that must sustain excessively heavy loads, potentially heightening their risk of a bone fracture. These findings may provide insight into more widespread upward trends in bone fragility and fracture risk among societies undergoing the energy balance transition.

## Introduction

In recent generations, societies around the world have experienced an energy balance transition characterized by decreased physical activity and increased dietary energy intake, resulting in a heightened tendency toward chronic positive energy balance^1^. Among the most salient outcomes of this transition has been a dramatic increase in the prevalence of obesity^2^, as well as related metabolic disorders including type II diabetes and cardiovascular disease^3,4^. In the United States, the transition began approximately a half-century ago and has since affected people of all ages, genders, and ethnicities, in both urban and rural areas^5–7^.

While it is unquestionable that the energy balance transition has had major consequences for general metabolism and health, for many systems of the body impacts of the transition are not fully understood. The skeletal system is a good example. Among the most critical components of skeletal health are bone mass and strength, defined here, respectively, as the quantity of bone tissue within skeletal elements and structural properties of skeletal elements that determine their ability to resist deformation and fracture during mechanical loading. Bone mass and strength are both well documented to be influenced by physical activity, diet, and obesity^8,9^, making it likely that average mass and strength have been affected in some way by the energy balance transition. By extension, susceptibility to osteopenia and osteoporosis has also likely been affected. Nevertheless, the precise ways in which the transition has influenced skeletal health are difficult to predict, as recent changes in physical activity, diet, and obesity levels might be expected to have different and complex effects.

In terms of physical activity, reduced activity levels would be expected to result in diminished bone mass and strength. Numerous lines of evidence indicate that bones have the capacity to adjust their size and structure in response to the mechanical loads they experience throughout life^8,10,11^. Typically, skeletal loads engendered by physical activity promote net bone formation and development and maintenance of bones that are well built to resist deformation and fracture^12^, whereas limited loading due to inactivity can lead to more slender, light bones that are prone to breaking^13^. Thus, given recent declines in physical activity, it has frequently been hypothesized that the energy balance transition has negatively affected skeletal health^14–18^.

In terms of diet, increased energy availability due to dietary changes could be expected to result in enhanced bone mass and strength. Bone is a metabolically expensive tissue and sufficient dietary energy intake is necessary to develop and maintain large, strong bones^19,20^. The importance of sufficient energy availability for skeletal health is underscored by the high prevalence of osteopenia, osteoporosis, and bone fractures among individuals who have experienced long periods of severe dietary caloric restriction^21–23^. In addition, among many societies, the energy balance transition has been associated with increases in average stature, suggesting that greater energy availability has generally promoted skeletal anabolism^24^. Also, experiments with animal models have found that energy-dense diets have the potential to increase bone mass and strength relative to standard laboratory diets^25–27^. Thus, from a dietary perspective, it is possible that the energy balance transition has had beneficial consequences for bone mass and strength.

In terms of the increased prevalence of obesity, both positive and negative effects on the skeleton might be expected. Studies of people in the United States and other post-industrial societies have shown that obesity is often associated with enhanced bone mass and strength, even in non-weight-bearing elements such as the radius^28–32^. Such evidence is consistent with the idea that greater energy availability generally promotes skeletal anabolism. Importantly, however, among people with obesity, increases in bone mass and strength are not necessarily commensurate with increases in body weight^28^. As a result, even with enhanced bone structure, many people with obesity may ultimately subject their bones to relatively higher mechanical stresses (i.e., higher loads relative to bone strength), potentially increasing their risk of a bone fracture, especially during a traumatic event such as a fall^28,32–34^. Therefore, when considering the effects of the energy balance transition on the skeleton, it is important to examine changes in bone mass and strength per se, as well as changes in bone properties relative to changes in body weight.

Here, we investigate the effects of the energy balance transition on bone mass and strength by focusing on the Indigenous peoples of New Mexico in the southwestern United States. The Indigenous peoples of New Mexico are an ideal case study because they are a rare example of a group for which it is possible to compare skeletal parameters between individuals living prior to and after the start of the energy balance transition. Concentrating on a particular group living in a specific region limits potential confounding effects of genetic and geographical influences on bone properties^35,36^. For this study, to assess pre-transition bone mass and strength, data were analyzed from a large sample of archaeological skeletal remains of adults who lived prior to the mid-nineteenth century. These individuals had physically active lifestyles and diets consisting of a mix of locally grown and wild foods. To assess post-transition skeletal mass and strength, we analyzed computed tomography (CT) scans of a large sample of modern-day adults. The lifestyles of these individuals in many ways reflect those of the current broader United States population, and those of many post-industrial societies in general, with many people engaging in low levels of physical activity and consuming market-based, energy-dense diets, resulting in chronic positive energy balance. Consequently, a comparison between New Mexico’s present and past Indigenous peoples has the potential to provide insights into more widespread effects of the energy balance transition on bone mass and strength.

## Methods

### Study samples

The sample of people living prior to the energy balance transition consisted of individuals who were members of what is today commonly called Pecos Pueblo, a large village occupied continuously between approximately the 1300s and early 1800s CE^37,38^. Pecos Pueblo is located in what is now north-central New Mexico in the valley of the Pecos River. Members of Pecos Pueblo are believed to have been speakers of the Towa language, and their living descendants are members of the Pueblo of Jemez located roughly 100 km away, where people also speak Towa. In the Towa language, Pecos Pueblo is referred to as P`ǽ kilâ, which translates to “the place above the water,” and the people of P`ǽ kilâ are referred to as the P`ǽ kish. During its time of occupation, Pecos Pueblo grew to become one of the most populous Indigenous villages and important trade centers in what is now the southwestern United States.

The members of Pecos Pueblo were subsistence farmers who grew mostly maize, beans, and squash^37,38^. They also hunted, gathered, and traded for wild foods^37,38^. Studies of living Indigenous peoples who subsist on farming supplemented by foraging have demonstrated that this way of life is physically demanding and very energetically costly^39^. In addition, studies of contemporary Indigenous peoples with diets similar to those at Pecos Pueblo have shown that the combination of foods consumed is high in nutritional quality and beneficial for metabolic health^40,41^. Thus, it is reasonable to assume that Pecos Pueblo members were at low risk of experiencing chronic positive energy balance and obesity. This assumption is also supported by historical anthropometric data indicating that obesity used to be rare among New Mexico’s Indigenous peoples prior to the energy balance transition^42,43^.

In the early twentieth century, archaeologists conducted extensive excavation of Pecos Pueblo and unearthed the skeletal remains of more than 1000 members, which were then sent to museums and academic institutions for research^44,45^. Because of the exceptionally large size and good preservation of the Pecos Pueblo human skeletal sample, it became one of the most studied collections by anthropologists interested in the biology of past peoples^45^. Among the many studies conducted were multiple analyses of bone mass and strength^46–49^. In 1999, however, following decades of struggle by Indigenous peoples against disrespectful treatment of their deceased ancestors, as well as passage of the Native American Graves Protection and Repatriation Act by the United States government, the skeletal remains of the members of Pecos Pueblo were reclaimed and reburied by the descendant community of the Pueblo of Jemez.

For this study, we re-analyzed data collected prior to the reburial of the Pecos Pueblo skeletal remains; thus, no remains were disturbed for the specific purpose of conducting the analyses reported here. Data were available from 126 adults, half females and half males^46–49^. Sex determination was based on dimorphic characteristics of the pelvis and cranium^50^. Age at death was estimated based on age-related changes of the pubic symphysis of the pelvis, dental wear, and cranial suture closure^50^. All individuals were estimated to be aged 18 years or older. Stature was estimated based on the maximum length of the femur using sex-specific predictive equations appropriate for Indigenous peoples of the southwestern United States^51^. Skeletal bi-iliac (maximum pelvic) breadth data were available from 33 individuals^52^, which were used to estimate living bi-iliac breadth using sex-specific predictive equations applicable to archaeological human remains^53^. Body weight was estimated based on skeletal frame size (SFS) using one of two types of sex-specific predictive equations. First, for individuals with available bi-iliac breadth data, body weight was estimated based on the combination of stature and living bi-iliac breadth using equations developed with a large reference sample of people with moderate body weights^54^. Second, body weight was estimated based on femoral head supero-inferior breadth, again using equations developed with a large reference sample of people with moderate body weights^55^. Here, a “moderate” body weight refers to a weight corresponding to a body mass index (BMI; calculated as weight in kg divided by stature in m^2^) of between 18.5 and 24.9. In conventional terminology (e.g., that used by the World Health Organization^56^), a body weight corresponding to this BMI range would be referred to as “healthy” or “normal”. However, these terms can be misleading given that BMI is an imperfect measure of overall health^57,58^, and in societies such as the contemporary United States population, most people have BMIs above this range^2,6^.

The modern-day sample of people consisted of Indigenous adults included in the New Mexico Decedent Image Database (NMDID), a collection of high-resolution full-body CT scans of more than 15,000 New Mexicans with associated demographic, lifestyle, and health information^59^. CT scans and associated information in the NMDID are from people who died between 2010 and 2017 and whose deaths were investigated by the New Mexico Office of the Medical Investigator. All CT scans and associated information are de-identified, and the associated information is derived from interviews with the next of kin of the decedent and the death investigation.

We included all individuals in the NMDID who met five criteria. First, all individuals were documented as being members of one or more of the 23 sovereign Indigenous nations of New Mexico, which include 19 Pueblo nations, 3 Apache nations, and the Diné (Navajo) Nation. Second, all individuals were aged 18 years or older at the time of their death. Third, none of the individuals’ bodies were markedly decomposed at the time of the medical investigator’s examination. Fourth, for all individuals, stature and body weight were measured by the medical investigator. For each individual, data on stature and body weight were used to calculate BMI and determine whether they were overweight (25 ≤ BMI < 30) or obese (BMI ≥ 30) based on World Health Organization standards^56^. Fifth, individuals were included only if their skeletons were observed in the CT scan images to be adequately intact to measure bone mass and strength (e.g., no amputations or skeletal fractures that hindered data collection). The final sample consisted of 212 individuals, 60 females and 152 males.

Multiple lines of evidence indicate that lifestyles varied among individuals in the modern-day sample. First, information on the general location of decedents’ addresses at the time of death was available for 192 individuals, 51% of which lived in a metropolitan area and 49% of which lived in more rural regions. Although all individuals in the sample had some access to the market economy, it is reasonable to assume that the diets of people living in metropolitan areas were somewhat more dependent on market foods and drinks. Second, information on socioeconomic status was available for 85 individuals, of which 6%, 55%, and 39% of people were categorized as having high, middle, and low socioeconomic status, respectively. Third, information on occupation was available for 90 individuals, each of which we assigned an estimate of occupational physical activity intensity based on a previously published classification scheme^60^. Among individuals with a documented occupation, 62% of people had jobs that were classified as sedentary or requiring light amounts of physical activity (e.g., sales occupations, food service occupations, teaching) and 38% had jobs involving moderate to large amounts of physical activity (e.g., construction, manufacturing, farming).

Among individuals in the modern-day sample, there was a high prevalence of overweight and obesity, indicating that many people experienced chronic positive energy balance. Among women, 33% and 38% of individuals were overweight or obese, respectively. Among men, 38% and 32% of individuals were overweight or obese, respectively. These levels of overweight and obesity are similar to estimates for the entire current United States adult population^6^, as well as for many other societies undergoing the energy balance transition^2^. In addition, many individuals in the sample were documented as having metabolic disorders commonly associated with obesity. Specifically, 20% of all individuals, and 27% of individuals aged 40 years or older, were known to have been diagnosed with hypertension, cardiovascular disease, and/or type II diabetes.

### Ethics declarations

This study was conducted in collaboration with the Tribal Historic Preservation Officer of the Pueblo of Jemez who is responsible for providing oversight, protection, and conservation of the tribal nation’s cultural resources, including those from Pecos Pueblo. Permission to analyze the CT scans of people in the modern-day sample was provided by each person’s next of kin to the developers of the NMDID. Additional collaborators on this study included multiple Indigenous students who analyzed the CT scans from the NMDID, all of whom are co-authors on this paper.

### Bone mass and strength measurements

To assess bone mass and strength, we analyzed diaphyseal cross-sectional geometry in the tibia^61^. The tibia was chosen for analysis for three reasons. First, tibial diaphyseal size and structure have consistently been shown to be influenced by physical activity levels^62–64^. Second, bone development in distal limb skeletal elements such as the tibia is more likely to be affected by energy availability than in proximal limb segment elements^65^. Third, data on tibial diaphyseal cross-sectional geometry from the members of Pecos Pueblo were available from prior research^46–49^.

Cross-sectional geometric analyses of limb bone diaphyses are based on modeling the bone as an engineering beam and calculating properties that reflect strength of the beam under loading^61^. In this study, we focused our analyses on properties that characterize strength of the tibial diaphysis in relation to the three types of loading it normally experiences during routine activities like walking and running: bending, axial compression, and torsion^66–68^. These properties included maximum and minimum second moments of area (*I*_max_ and *I*_min_), which describe diaphyseal resistance to bending around principal axes; cortical bone area (Ct.Ar), which describes diaphyseal resistance to axial loading; and polar second moment of area (*J*), which describes diaphyseal resistance to torsional and average bending loads. Ct.Ar, in addition to reflecting axial loading strength, is a measure of bone tissue quantity (i.e., bone mass).

Geometric properties were measured in the mid-diaphysis of the tibia, defined as half the bone’s articular length^46^. As described elsewhere^46,48,49^, for Pecos Pueblo members, properties were measured in 119 and 7 individuals from transverse cut sections and CT image slices of the mid-diaphysis, respectively. The 7 individuals measured using CT were aged 18 and 19 years and from an ontogenetic study of bone mass and strength^48^. Data on diaphyseal cross-sectional geometry acquired from cut sections and CT images have been shown to be comparable^69^. For each person in the modern-day sample, their full-body CT image stack was imported into Amira software and a 3D digital rendering of their full-body skeleton was generated to locate a tibia. The tibia was then digitally cropped out from the full-body skeleton using the Amira “Volume Edit” option with draw tools and outside cutting properties, and tibial articular length was measured using the 3D ruler tool. The cropped-out tibia was then saved as a separate CT image stack. Next, the tibial CT image stack was imported into ImageJ software and a 3D digital rendering of the bone was aligned longitudinally using the BoneJ plugin^70^. Finally, on the aligned tibia, the transverse CT image slice corresponding to the mid-diaphysis was selected and geometric properties were calculated using BoneJ. In BoneJ, bone was distinguished from non-bone in CT images using the half-maximum height thresholding method^71^.

When comparing diaphyseal cross-sectional geometric properties between individuals and samples, it is important to control for variation in body size, as taller and/or heavier people tend to have greater bone mass and strength^61,72^. From a mechanical perspective, the most relevant measure for scaling Ct.Ar is body weight since axial stress in a diaphysis is proportional to axial force^61^. Similarly, *I*_max_, *I*_min_, and* J* should be scaled by the product of body weight and bone length squared since bending and torsional stresses in a diaphysis are proportional to bending/torsional force times its moment arm length squared^61^. Based on this reasoning, for this study, we adopted two different strategies of scaling tibial mid-diaphyseal cross-sectional geometric properties, which enabled us to separately examine changes over time in bone mass and strength while simultaneously accounting for differences in body weight between people living before and after the start of the energy balance transition, as well as changes in bone properties specifically.

First, tibial mid-diaphyseal Ct.Ar was size standardized by dividing values by SFS-estimated body weights among Pecos Pueblo members and measured body weights among modern-day people. *I*_max_, *I*_min_, and* J* values were divided by the product of body weight (estimated or measured) and tibial articular length squared. Given the high prevalence of overweight and obesity among people in the modern-day sample, this strategy of scaling diaphyseal geometric properties using measured body weights inevitably conflates potential effects of the energy balance transition on the skeleton with its effects on body weight. As a result, high overweight and obesity levels in the modern-day sample have the potential to mask any increases in bone mass and strength if increases in bone properties were not proportional to increases in body weight. Nevertheless, from a mechanical standpoint, this conflation is logical and meaningful since the strength of a bone is relative to the loads it sustains.

Second, for each person in the modern-day sample, we calculated their SFS-estimated body weight based on their stature and living bi-iliac breadth using the same sex-specific predictive equations applied to Pecos Pueblo members^54^. Living bi-iliac breadth values were obtained by measuring skeletal bi-iliac breadth in the full-body CT scans using the 3D ruler tool in Amira, and then converting measured values to living bi-iliac breadths using sex-specific predictive equations applicable to full-body CT scans^55^. Then, we size standardized diaphyseal geometric properties a second time as described above, only instead of using measured body weights, we used SFS-estimated body weights. Although this scaling strategy may be less mechanically meaningful than using measured body weights, it provides a potentially informative heuristic for revealing differences in bone mass and strength per se in comparisons between people in the modern-day sample and Pecos Pueblo members, while still accounting for potential temporal changes in skeletal frame size. Importantly, any bone property differences detected between time periods using this scaling strategy for the modern-day sample should not be interpreted as being independent of group differences in body weight, as it does not eliminate any effects of overweight and obesity on bone mass and strength. Rather, it simply decreases the likelihood that changes in bone properties per se are obscured by the high overweight and obesity levels in the post-transition sample.

In addition, two geometric properties were calculated that do not require size standardization: percent cortical bone area (%Ct.Ar), which is the relative amount of total diaphyseal cross-sectional area taken up by cortical bone and thus a relative measure of bone tissue quantity, and the diaphyseal bending strength index (*I*_max_/*I*_min_), which is a relative measure of maximum diaphyseal bending strength^61^.

### Statistical analyses

**Figure 1:**
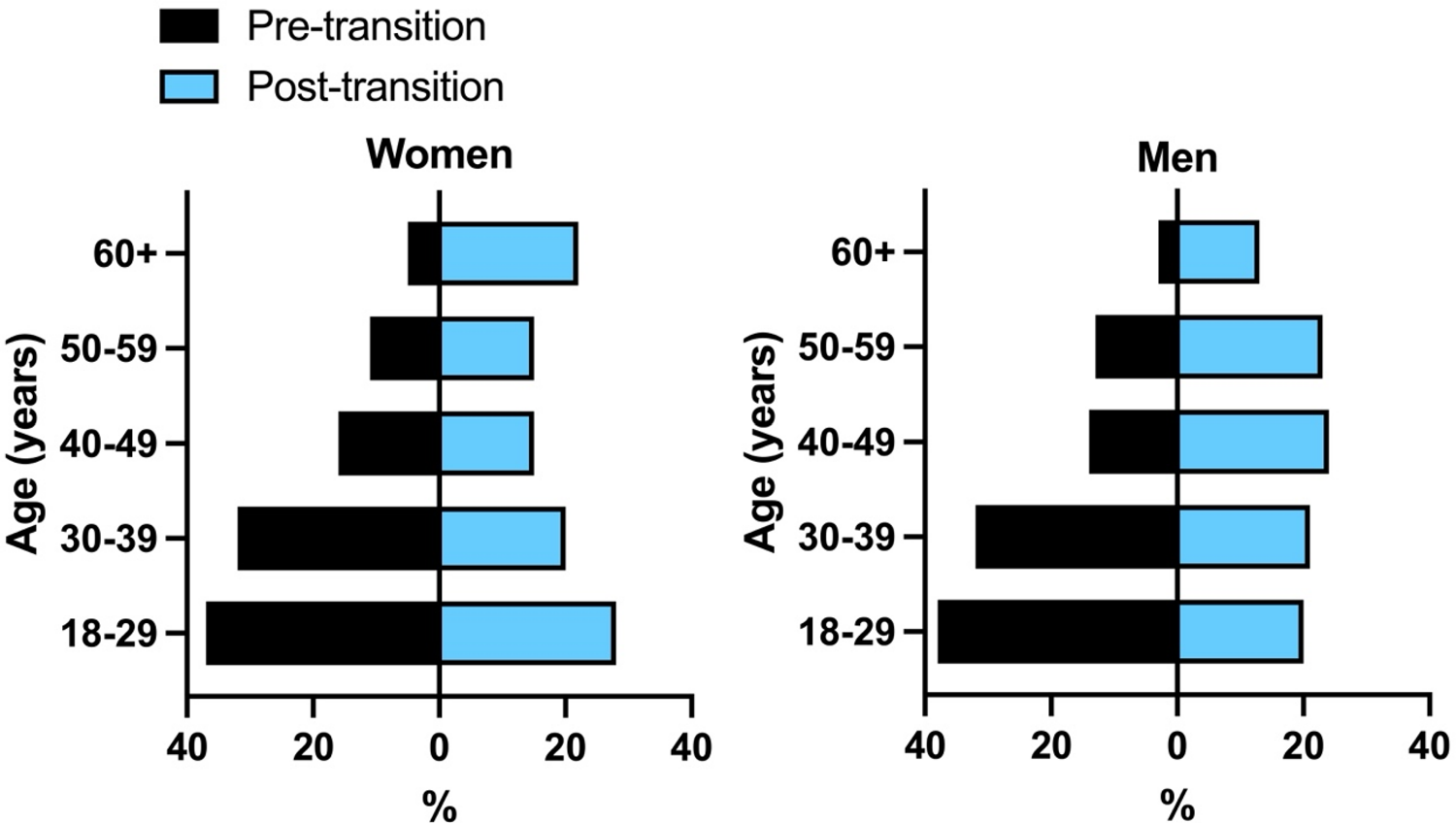
Age distributions in the samples of Indigenous women (*left*) and men (*right*) in New Mexico living before and after the start of the energy balance transition.

To test for differences in bone mass and strength properties between people living before and after the start of the energy balance transition, we used general linear models. Age was included as a covariate in the models because age is known to affect bone mass and strength^47,48,73^, and age composition differed somewhat between the pre- and post-transition samples. Specifically, though both samples were comprised mainly of young (aged 18–39 years) and middle-aged (aged 40–60 years) adults, the post-transition sample had a greater proportion of people aged 60 years and older (Fig. 1). In analyses of size-standardized bone properties, comparisons were made between three datasets: bone properties from pre-transition individuals scaled using SFS-estimated body weights; bone properties from post-transition individuals scaled using SFS-estimated body weights; and bone properties from post-transition individuals scaled using measured body weights. In these analyses, least squares means of bone properties from the three datasets were compared using Tukey HSD tests. We also used *t*-tests to compare stature and living bi-iliac breadth between pre- and post-transition individuals, as well as paired-samples *t*-tests to compare measured and SFS-estimated body weights among people in the post-transition sample. In addition, to investigate the effects of overweight and obesity on bone mass and strength among people in the modern-day sample, we used general linear models followed by Tukey HSD tests to assess differences in Ct.Ar and second moments of area, scaled using both SFS-estimated body weights and measured body weights, between overweight, obese, and non-overweight/obese individuals, with age included as a covariate. General linear models followed by Tukey HSD tests were also used to assess differences in %Ct.Ar and *I*_max_/*I*_min_ between overweight, obese, and non-overweight/obese modern-day individuals, with age included as a covariate. Women and men were analyzed separately. All analyses were performed using JMP Pro software. Statistical significance was judged using a 95% criterion (*p* ≤ 0.05). Descriptive statistics for body size and tibial attributes separated by sex and time period are reported in Table 1.

**Table 1:** Descriptive statistics for body size, tibial articular length, and unstandardized tibial mid-diaphyseal cross-sectional geometric properties among Indigenous women and men in New Mexico living before and after the start of the energy balance transition.

	Women		Men	
	Pre-transition	Post-transition	Pre-transition	Post-transition
Stature (cm)	149 ± 5	161 ± 9	161 ± 4	175 ± 8
Living bi-iliac breadth (cm)	27.6 ± 1.7	28.2 ± 2.2	27.7 ± 1.4	28.3 ± 1.7
SFS-estimated body weight (kg)	48.9 ± 5.7	59.6 ± 7.4	59.7 ± 5.4	69.2 ± 7.5
Measured body weight (kg)	N/A	77.3 ± 23.9	N/A	85.9 ± 21.9
Tibial articular length (mm)	307 ± 17	348 ± 22	338 ± 16	381 ± 22
*I*_max_ (mm^4^)	13,181 ± 3648	17,171 ± 5602	24,380 ± 6009	29,603 ± 7794
*I*_min_ (mm^4^)	5065 ± 1560	7787 ± 2475	8113 ± 2366	12,943 ± 3317
Ct.Ar (mm^2^)	207 ± 36	268 ± 52	284 ± 42	354 ± 50
*J* (mm^4^)	18,246 ± 4968	24,958 ± 7955	32,493 ± 7830	42,547 ± 10,584
%Ct.Ar (%)	61.0 ± 10.9	68.1 ± 9.9	64.5 ± 8.5	69.9 ± 5.8
*I*_max_/*I*_min_	2.7 ± 0.5	2.2 ± 0.3	3.1 ± 0.6	2.3 ± 0.4

Means are shown ± standard deviations.

*SFS* skeletal frame size.

## Results

### Effects of the energy balance transition on body size

**Figure 2:**
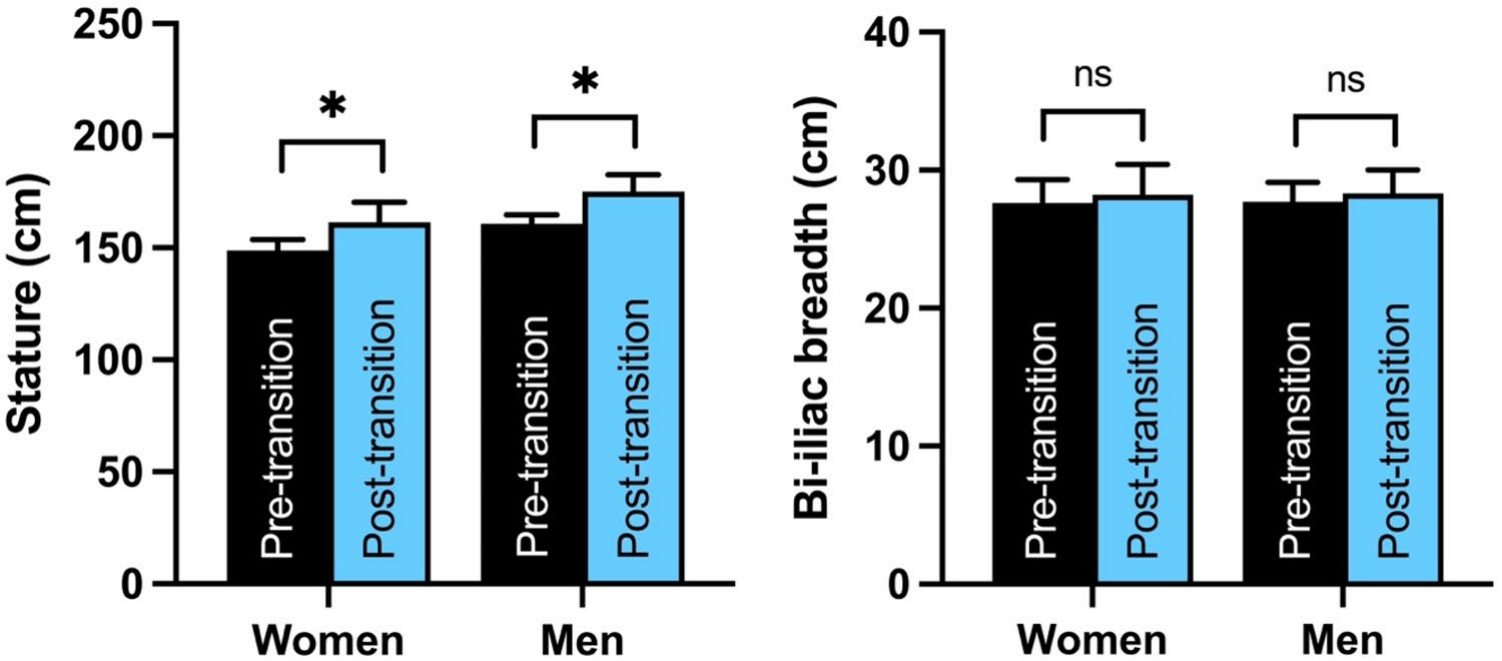
The energy balance transition has been associated with significant increases in stature (*left*) but not living bi-iliac breadth (*right*) among Indigenous women and men in New Mexico. Bars are means + standard deviations. Significance codes: not significant (ns), *p* < 0.0001 (*).

**Figure 3:**
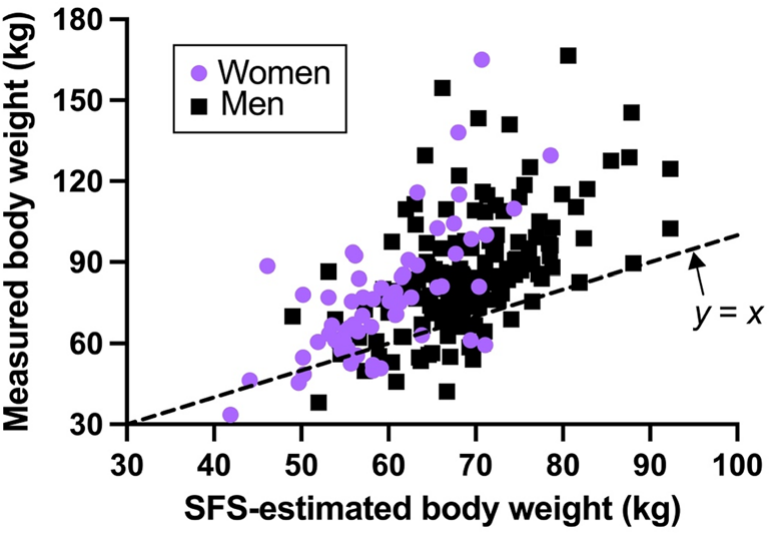
Among Indigenous women and men in New Mexico living after the start of the energy balance transition, measured body weights (*y* axis) were significantly higher (*p* < 0.0001 for both sexes) than body weights estimated based on skeletal frame size (SFS) (*x* axis).

Indigenous women and men in New Mexico living after the start of the energy balance transition were, on average, 8% and 9% taller, respectively, than women and men living prior to the transition (*p* < 0.0001 for both sexes; Fig. 2). Bi-iliac breadth was not significantly different in either sex between people living before and after the transition. Among people living after the transition, the measured body weights of women and men were, on average, 30% and 24% heavier, respectively, than their SFS-estimated body weights (*p* < 0.0001 for both sexes; Fig. 3).

### Effects of the energy balance transition on bone mass and strength

**Figure 4:**
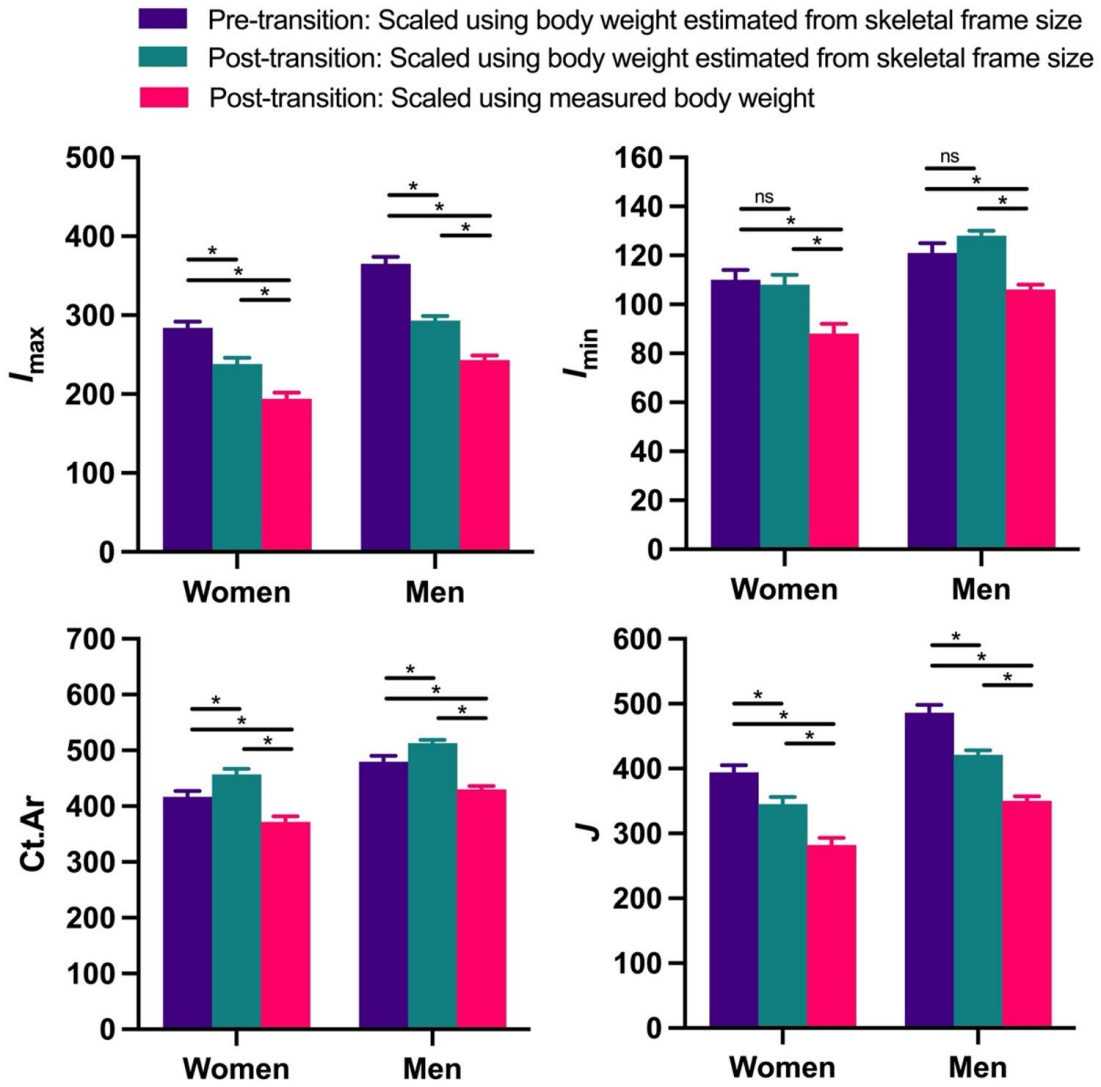
Among Indigenous women and men in New Mexico, the energy balance transition has been associated with significant changes in bone mass and strength properties of the tibial mid-diaphysis, including maximum second moment of area (*I*_max_; *top left*), minimum second moment of area (*I*_min_; *top right*), cortical bone area (Ct.Ar; *bottom left*), and polar second moment of area (*J*; *bottom right*). All properties were size standardized prior to analysis. *I*_max_, *I*_min_, and *J* values were standardized by dividing by the product of body weight (estimated or measured) and tibial articular length^2^, and units in the figure are mm^4^/(kg·mm^2^)·10^5^. Ct.Ar values were standardized by dividing by body weight (estimated or measured), and units in the figure are mm^2^/kg·10^2^. Bars are least squares means + standard errors from tests that controlled for age. Significance codes: not significant (ns), *p* < 0.05 (*).

Among people living after the start of the energy balance transition, when bone mass and strength properties of the tibial mid-diaphysis were size standardized using measured body weights, all properties were significantly lower than the standardized properties of people living prior to the transition (Fig. 4). Among women living after the transition, size-standardized *I*_max_, *I*_min_, Ct.Ar, and *J* were, on average, 32%, 20%, 11%, and 28% lower, respectively, than among women living prior to the transition (*p* < 0.0001, *p* < 0.0001, *p* < 0.01, and *p* < 0.0001, respectively). Among men living after the transition, these same size-standardized properties were, on average, 33%, 12%, 10%, and 28% lower, respectively, than among men living before the transition (*p* < 0.0001, *p* < 0.01, *p* < 0.001, and *p* < 0.0001, respectively).

When bone mass and strength properties of people living after the start of the transition were size standardized using their SFS-estimated body weights, their size-standardized *I*_max_ values remained significantly lower than those of people living before the transition; on average, by 16% among women (*p* < 0.001; Fig. 4) and 20% among men (*p* < 0.0001). Similarly, size-standardized *J* values remained significantly lower among people in the post- versus pre-transition samples, by an average of 12% among women (*p* < 0.01) and 13% among men (*p* < 0.0001). In contrast, however, among people living after the transition, size-standardized Ct.Ar was significantly higher than among people in the pre-transition sample, by an average of 9% among women (*p* = 0.017) and 6% among men (*p* = 0.018). Size-standardized *I*_min_ values did not differ significantly in either sex between people living before and after the transition.

**Figure 5:**
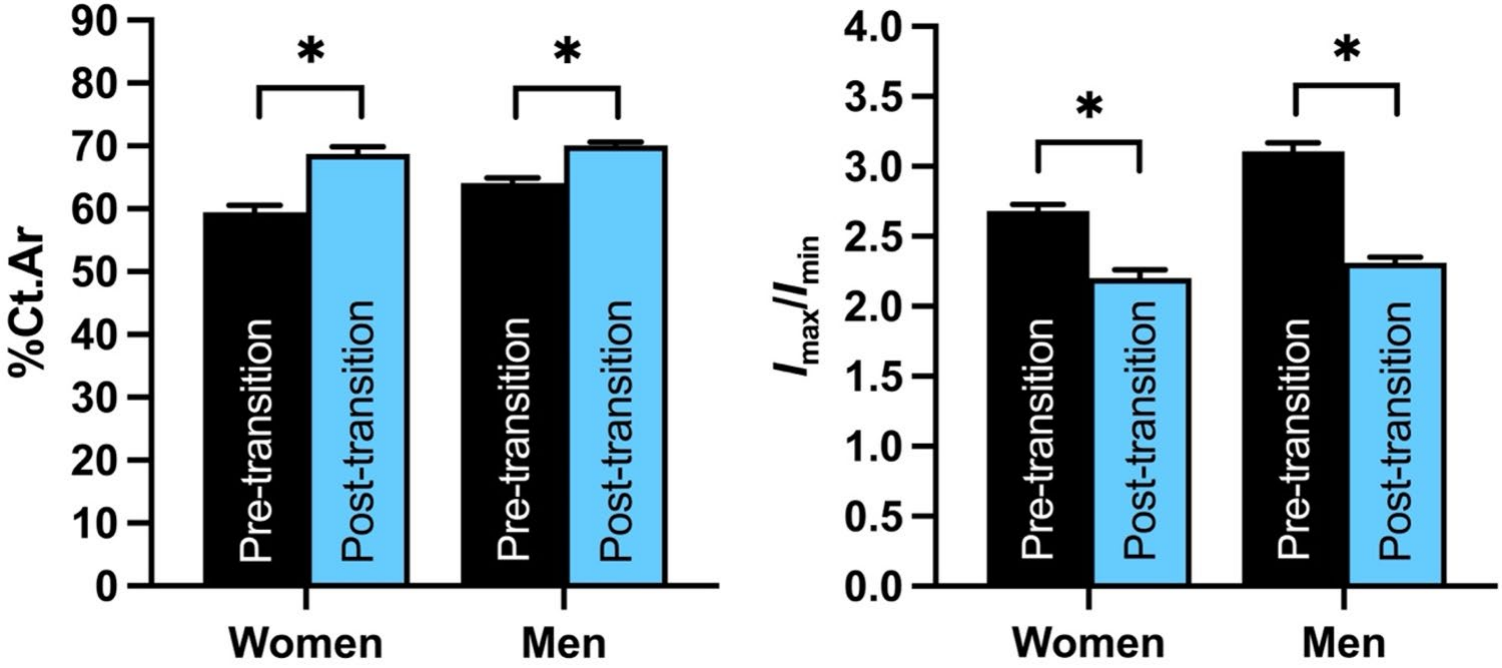
Among Indigenous women and men in New Mexico, the energy balance transition has been associated with significant increases in percent cortical bone area (%Ct.Ar; *left*) and decreases in the bending strength index (*I*_max_/*I*_min_; *right*) in the tibial mid-diaphysis. Bars are least squares means + standard errors from tests that controlled for age. Significance code: *p* < 0.0001 (*).

Both of the bone properties analyzed that did not require size standardization differed significantly between people living before and after the start of the transition (Fig. 5). Among women and men living after the transition, %Ct.Ar was, on average, 17% and 9% higher, respectively, than among women and men living before the transition (*p* < 0.0001 for both sexes), whereas *I*_max_/*I*_min_ values were, on average, 18% and 26% lower, respectively, than among women and men living prior to the transition (*p* < 0.0001 for both sexes).

### Effects of overweight and obesity on bone mass and strength

**Figure 6:**
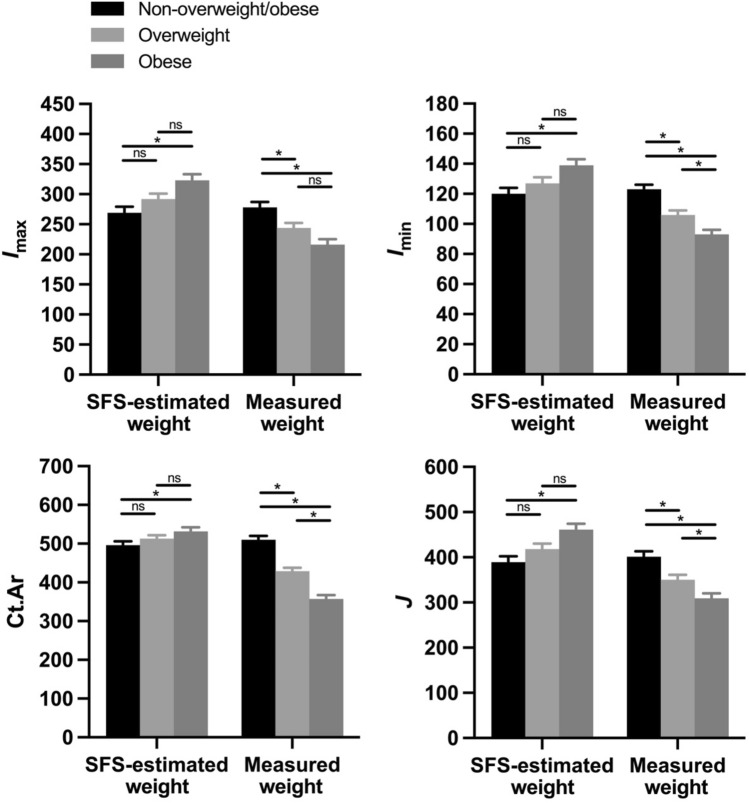
Among men in the modern-day sample, the presence of overweight and obesity significantly affected bone mass and strength properties of the tibial mid-diaphysis, including maximum second moment of area (*I*_max_; *top left*), minimum second moment of area (*I*_min_; *top right*), cortical bone area (Ct.Ar; *bottom left*), and polar second moment of area (*J*; *bottom right*). All properties were size standardized prior to analysis using both measured body weight and body weight estimated based on skeletal frame size (SFS). *I*_max_, *I*_min_, and *J* values were standardized by dividing by the product of body weight (estimated or measured) and tibial articular length^2^, and units in the figure are mm^4^/(kg·mm^2^)·10^5^. Ct.Ar values were standardized by dividing by body weight (estimated or measured), and units in the figure are mm^2^/kg·10^2^. Bars are least squares means + standard errors from tests that controlled for age. Significance codes: not significant (ns), *p* < 0.05 (*).

Among men living after the start of the energy balance transition, when bone mass and strength properties were size standardized using SFS-estimated body weights, all bone properties were significantly greater among obese individuals than non-overweight/obese individuals (Fig. 6). Specifically, relative to non-overweight/obese people, size-standardized *I*_max_, *I*_min_, Ct.Ar, and *J* were, on average, 20%, 16%, 7%, and 19% higher, respectively, among obese individuals (*p* < 0.001, *p* < 0.01, *p* < 0.05, and *p* < 0.001, respectively). However, when bone mass and strength properties were standardized using measured body weights, all bone properties were significantly lower among overweight and obese individuals compared to non-overweight/obese individuals. Specifically, relative to non-overweight/obese people, size-standardized *I*_max_, *I*_min_, Ct.Ar, and *J* were, on average, 12%, 14%, 16%, and 13% lower, respectively, among overweight individuals (*p* < 0.05, *p* < 0.001, *p* < 0.0001, and *p* < 0.01, respectively), and 22%, 24%, 30%, and 23% lower, respectively, among obese individuals (*p* < 0.0001 for all comparisons).

**Figure 7:**
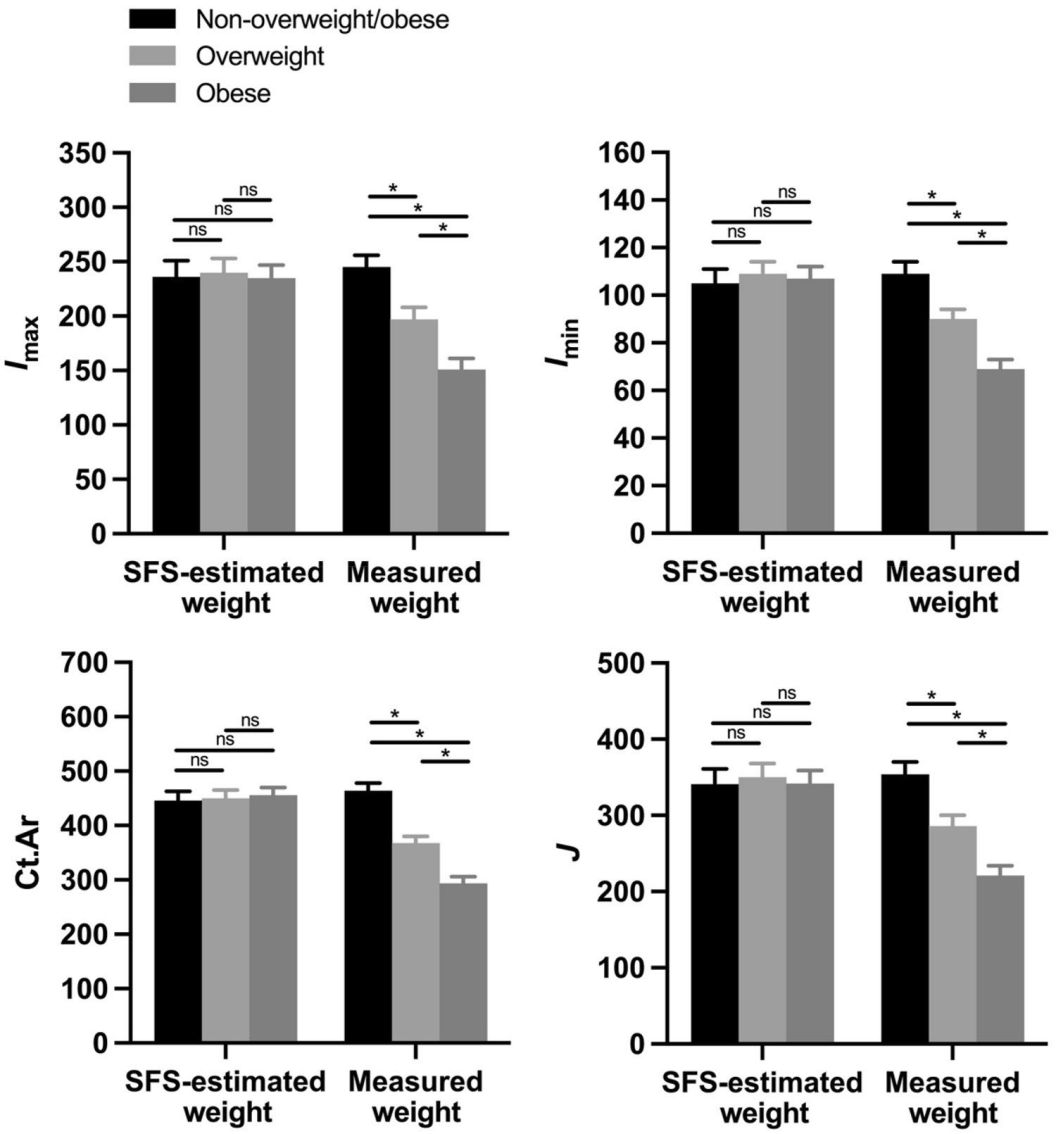
Among women in the modern-day sample, the presence of overweight and obesity significantly affected bone mass and strength properties of the tibial mid-diaphysis, including maximum second moment of area (*I*_max_; *top left*), minimum second moment of area (*I*_min_; *top right*), cortical bone area (Ct.Ar; *bottom left*), and polar second moment of area (*J*; *bottom right*). All properties were size standardized prior to analysis using both measured body weight and body weight estimated based on skeletal frame size (SFS). *I*_max_, *I*_min_, and *J* values were standardized by dividing by the product of body weight (estimated or measured) and tibial articular length^2^, and units in the figure are mm^4^/(kg·mm^2^)·10^5^. Ct.Ar values were standardized by dividing by body weight (estimated or measured), and units in the figure are mm^2^/kg·10^2^. Bars are least squares means + standard errors from tests that controlled for age. Significance codes: not significant (ns), *p* < 0.05 (*).

Among women in the modern-day sample, when bone mass and strength properties were size standardized using SFS-estimated body weights, none of the properties differed significantly between overweight, obese, and non-overweight/obese individuals (Fig. 7). However, similar to men, when bone mass and strength properties were standardized using measured body weights, all bone properties were significantly lower among overweight and obese individuals compared to non-overweight/obese individuals. Specifically, relative to non-overweight/obese people, size-standardized *I*_max_, *I*_min_, Ct.Ar, and *J* were, on average, 20%, 17%, 21%, and 19% lower, respectively, among overweight individuals (*p* < 0.01, *p* < 0.05, *p* < 0.0001, and *p* < 0.01, respectively), and 38%, 37%, 37%, and 38% lower, respectively, among obese individuals (*p* < 0.0001 for all comparisons).

Among both men and women, neither of the bone properties analyzed that did not require size standardization (%Ct.Ar, *I*_max_/*I*_min_) differed significantly between overweight, obese, and non-overweight/obese individuals.

## Discussion

The goal of this study was to gain insight into the effects of the energy balance transition on bone mass and strength. To accomplish this goal, we focused on the Indigenous peoples of New Mexico, a rare case of a group for which bone mass and strength properties could be compared between individuals living prior to and after the start of the transition. We examined two alternative possibilities about how skeletal health has been affected by the transition. A common hypothesis is that bone mass and strength have declined because of the transition, primarily due to reductions in physical activity levels^14–18^. Alternatively, the transition has potentially improved skeletal health, since increased dietary energy intake and greater energy availability may enable people to devote more energy to developing and maintaining large, strong bones. To assess whether bone mass and strength have decreased or increased over time, we analyzed cross-sectional geometric properties of the mid-diaphysis of the tibia. Bone properties were standardized for body size in two different ways, using either SFS-estimated body weights or measured body weights for people in the modern-day sample, which enabled us to separately examine temporal changes in bone mass and strength specifically, as well as changes in bone properties while taking into account the high body weights of many people in the modern-day sample, respectively. Overall, our findings suggest that the energy balance transition has led to major decreases in bone strength, especially bone strength relative to measured body weight; however, we also found evidence that bone mass has increased over time.

Our key finding suggesting that the energy balance transition has led to an increase in bone mass is that when Ct.Ar was size standardized using SFS-estimated body weights, people living after the start of the energy balance transition had higher values than people living prior to the transition. Although we also found that when Ct.Ar was standardized using measured body weights among people in the modern-day sample, their Ct.Ar values were actually lower than among people living before the transition, this difference between samples conflates the effects of the transition on bone mass with its effects on body weight. Standardizing Ct.Ar using SFS-estimated body weights made it possible to better identify the effects of the transition on bone mass specifically*.* Ultimately, our results suggest that people living after the transition had greater bone tissue quantity than prior to the transition. Additional support for this conclusion was our finding that %Ct.Ar, a relative measure of bone mass that does not require size standardization, was higher among the post- than pre-transition sample. That people in the modern-day sample were also taller than in the pre-transition sample is another indication that the transition generally promoted skeletal anabolism. The observed similarity in bi-iliac breadth between people in the pre- and post-transition samples is consistent with the hypothesis that this trait is less phenotypically plastic than stature, probably due to obstetric and climatic constraints^74^.

Increases in bone tissue quantity following the start of the energy balance transition might be expected to translate directly into increases in diaphyseal strength in bending, axial loading, and torsion. However, this would only be true if the size of the outer perimeter of the diaphysis was unaffected or enlarged by the transition. If the diaphysis were to become more slender over time, then its strength in bending and torsion could decrease even as the amount of bone tissue increased^14,61^. This was precisely our finding. Regardless of whether *I*_max_ and *J* were size standardized using measured or SFS-estimated body weights among people in the modern-day sample, values were, on average, lower among people living after than before the start of the transition, indicating increased diaphyseal slenderness and decreased bending and torsional strength over time. Bone strength differences were greater between pre- and post-transition samples when *I*_max_ and *J* were standardized using measured body weights among people in the modern-day sample, highlighting that declines in relative bone strength have been compounded by increases in body weight over time. Indeed, the fact that even Ct.Ar when standardized using measured body weights was lower among the modern-day than pre-transition sample indicates that diaphyseal axial loading strength has decreased relative to the loads sustained during life. Thus, despite increases in bone tissue quantity over time, from a mechanical standpoint, the energy balance transition has had clear negative consequences for skeletal health. Many people today have more slender, weaker bones that must sustain heavier loads (due to greater body weight), potentially increasing the likelihood of a bone fracture, especially during a traumatic event such as a fall^28,32–34^. Interestingly, our finding that increases in bone mass have not inevitably led to increases in bone strength may help explain the so-called “calcium paradox”, the observation that many countries, including the United States, with high osteoporotic fracture rates also have generally high levels of dietary calcium intake^75,76^.

Although this is a retrospective study and we thus cannot test causation, it is important to consider the factors most likely to have been responsible for the changes in bone mass and strength following the start of the energy balance transition, particularly physical activity, diet, and obesity. Today’s high levels of obesity have certainly contributed to declines in bone strength relative to body weight, as have changes in physical activity and diet to the extent that these factors influence obesity risk. This was clearly demonstrated by our finding that among people in the post-transition sample, bone mass and strength properties were all significantly lower among overweight and obese individuals than non-overweight/obese individuals when bone properties were size standardized using measured body weights. But what about the effects of physical activity, diet, and obesity on bone property changes per se? Although all bone properties considered in this study have the potential to be affected by physical activity, diet, and obesity^25–27,77–79^, there are at least two reasons to suspect that the observed decreases in second moments of area were primarily due to reductions in physical activity. First, longitudinal studies among humans and experiments with animal models have shown that greater physical activity is consistently associated with enhancement of *I*_max_, *J*, and *I*_max_/*I*_min_, even more consistently than with increases in Ct.Ar^62,80,81^. Thus, it is reasonable to expect that second moments of area would be especially negatively affected by reduced physical activity. Second, there is little evidence from humans or animal models that energy-dense diets or obesity have direct negative effects on second moments of area. On the contrary, experiments with animal models have found that energy-dense diets have the potential to enhance second moments of area relative to standard laboratory diets^25–27^, and evidence from humans indicates that obesity is often associated with greater second moments of areas (without accounting for body weight variation)^28–32^. Indeed, in this study, among men in the modern-day sample, we found that people with obesity had higher *I*_max_, *I*_min_, and *J* values than non-overweight/obese people when bone properties were standardized using SFS-estimated body weights. As for the observed increase in bone mass following the start of the transition, reductions in physical activity were unlikely a major influence, given abundant evidence that mechanical loading is generally good for skeletal health^8,10,11^. In principle, Ct.Ar should be especially sensitive to variation in dietary caloric intake and energy availability, assuming the energetic cost of developing and maintaining skeletal elements is related to the amount of bone tissue that must be supported^19,20^. Consistent with this idea, studies of people experiencing chronic dietary caloric limitation have frequently documented decreases in Ct.Ar without concomitant decreases in diaphyseal circumference, implying that energy limitation affects bone tissue quantity more than second moments of area^82–85^. Also, in an experiment with mice, animals fed an energy-dense diet were found to have increased Ct.Ar in the femoral mid-diaphysis compared to animals fed a standard laboratory diet, but second moments did not differ between groups^27^. Thus, it is not unreasonable to speculate that the observed increase in bone mass after the start of the transition was primarily due to greater dietary caloric intake and energy availability. Importantly, however, not all our results are completely consistent with this hypothesis. In particular, if the increase in bone mass was primarily due to greater energy availability, it is unclear why obesity was not associated with greater Ct.Ar among women in the modern-day sample. More research is required to better understand the roles of physical activity, diet, obesity, and potentially other factors in producing the changes over time in bone mass and strength documented here.

Finally, the similarity between the lifestyles of the people in our modern-day sample and those of the current broader United States population, as well as those of many other post-industrial societies, suggests that the Indigenous peoples of New Mexico are almost certainly not alone in having experienced negative effects of the energy balance transition on skeletal health. In all likelihood, declines in bone strength have been a widespread phenomenon. However, some caution is required in extrapolating the results of this study to all other countries and populations undergoing the transition. Importantly, not all societies going through the transition have experienced decreases in physical activity as dramatic as those in the United States^86^. Among such societies, increased risk of chronic positive energy balance has so far been driven primarily by dietary changes^87–89^. If reductions in bone strength in the United States have been due in large part to decreased physical activity, then societies that have maintained relatively high levels of activity may not have experienced the same declines in bone strength. Nevertheless, in such societies, even if bone strength properties have not been greatly affected by the transition, increases in body weight have presumably led many individuals to subject their bones to heavier loads, potentially increasing their risk of a bone fracture, especially during a fall or other traumatic event^28,32–34^. Ultimately, additional comparisons similar to those of the present study are needed to assess possible global variation in the effects of the energy balance transition on skeletal health.

## Data Availability

The data generated and used in this study will be made available from the corresponding author upon request.
